# Henoch-Schönlein purpura following COVID-19 vaccine in a child: a case report

**DOI:** 10.1186/s13052-022-01351-1

**Published:** 2022-09-02

**Authors:** Francesca Casini, Vittoria Carlotta Magenes, Marina De Sanctis, Maurizio Gattinara, Marco Pandolfi, Stefano Cambiaghi, Gian Vincenzo Zuccotti, Valentina Fabiano

**Affiliations:** 1 Pediatric Department, “Vittore Buzzi” Children’s Hospital, Milan, Italy; 2 Primary Care Pediatrician, ASST Ovest Milan, ATS MILAN, Milan, Italy; 3 Pediatric Rheumatology Department, ASST Gaetano Pini-CTO, Milan, Italy; 4 Unit of Pediatric Dermatology, Department of Pathophysiology and Transplantation, Maggiore Policlinic Hospital, IRCCS Ca’ Granda Foundation, Università Di Milano, Milan, Italy; 5 Department of Biomedical and Clinical Sciences, Università Di Milano, Milan, Italy

**Keywords:** Henoch-Schönlein purpura (HSP), Case report, COVID-19 vaccine, Pfizer-BioNTech BNT16B2b2 mRNA vaccine, IgA-mediated vasculitis

## Abstract

**Background:**

Henoch-Schönlein purpura (HSP) is an IgA-mediated small vessel vasculitis, typical of childhood. It’s a self-limiting disease and it affects different systems. HSP is characterized by dermatological, abdominal, joint and renal clinical manifestations. This condition usually occurs upon infections, mainly upper respiratory tract ones, medications, vaccinations and malignancies.

**Case presentation:**

We describe the case of a 11 year-old girl who developed a urticarial rash 12 days after the first dose of Pfizer-BioNTech BNT16B2b2 mRNA vaccine and a clear picture of Henoch Schönlein purpura 5 days after administration of the second dose of the same vaccine.

**Conclusion:**

To our knowledge, this is the first description of a pediatric patient with Henoch-Schönlein purpura occurring in association with vaccination against COVID-19.

## Background

Henoch-Schönlein purpura (HSP) is a systemic small vessel vasculitis that commonly occurs in children. It is characterized by the deposition of immunoglobulin A (IgA)-based immune complexes on the walls of small blood vessels. The incidence rate of HSP is around 13–20 cases per 100,000 children under 17 years old. This condition can be triggered by several factors, such as infections, medications, circulating immune complexes and environmental insults. HSP is a complex disease, caused by several genetic and environmental factors. HSP is characterized by non-thrombocytopenic palpable purpura, mostly located on the lower extremities and buttocks, arthralgia/arthritis, bowel angina, and haematuria/proteinuria. Treatment is supportive as the disease course is generally benign and self-limited [1]. Progressive renal impairment, bowel perforation, and central nerve system involvement are the major morbidities of HSP. When these conditions present, aggressive therapies with steroid and/or immunosuppressants are indicated. The HSP diagnosis is based on the revised criteria developed by the European League Against Rheumatism, the Pediatric Rheumatology International Trials Organization and the Pediatric Rheumatology European Society (EULAR/PRINTO/PRES) in 2010, which consists of mandatory and supportive criteria [2], (Table 1). The sensitivity of these criteria is 100% [1].

**Table 1:** Diagnostic criteria for Henoch-Schönlein purpura (HSP), as developed by EULAR/PRINTO/PRES [2]

***Criterion***	***Description***
**Mandatory criterion**	Purpura or petechiae with lower limb predominance
**Minimum 1 out of 4 criteria**	1. Diffuse abdominal pain with acute onset 2. Histopathology showing leukocytoclastic vasculitis or proliferative glomerulonephritis with predominant immunoglobulin A (IgA) deposits 3. Arthritis or arthralgia of acute onset 4. Renal involvement in the form of proteinuria or haematuria

Herein, we discuss the case of an 11-year-old HSP patient after the Pfizer-BioNTech BNT16B2b2 mRNA vaccine. The aim of our work is to report a possible association between a preceding COVID-19 vaccination and a first onset of HSP in a previously healthy child.

## Case presentation

A 11 year-old girl came to our observation for a 3-day history of rash all over the body. The rash had an acute onset and was bilaterally distributed, mainly on the face and limbs. It was also associated with mild oedema of the extremities. The mother reported that the child was not complaining of fever, diarrhea or haematuria. The girl only had 2 episodes of acute diffuse abdominal pain. Moreover, this was the first occurrence of such an episode. During the preceding six weeks the girl had no signs or symptoms of infectious diseases nor she had assumed any drugs. She received the first dose of the Pfizer-BioNTech BNT16B2b2 mRNA vaccine exactly 12 days prior to presenting the reported skin manifestation. She has never been positive for COVID-19 and she has no relevant medical antecedents. Moreover, her familiar medical history was unremarkable: no autoimmune or chronic pathologies were reported in her relatives.

**Fig. 1:**
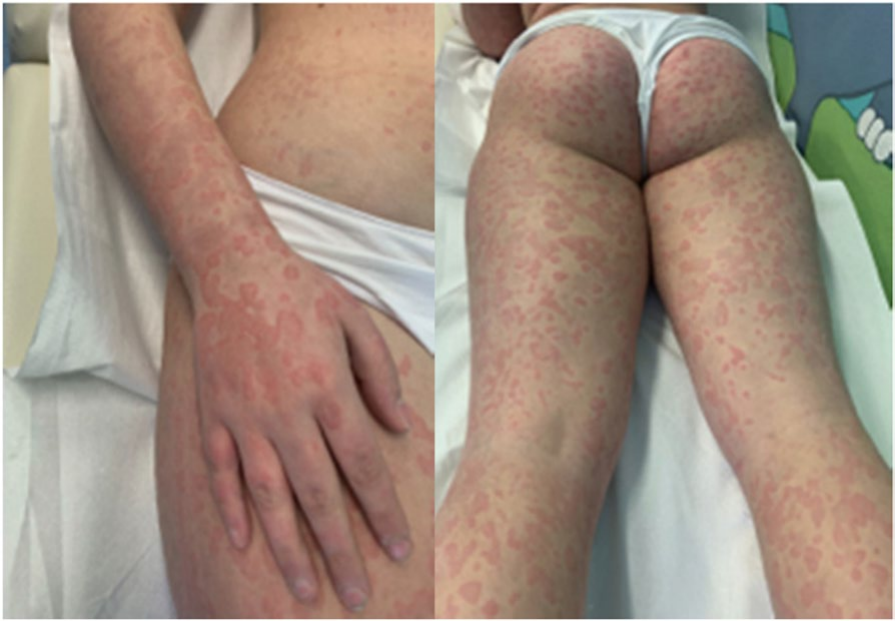
The figured urticarial rash appeared on her limbs and on her abdomen after the first dose of Pfizer-BioNTech BNT16B2b2 mRNA vaccine

At the visit, the patient was alert, conscious and well hydrated. Her vital signs were normal for age. At the skin examination, she showed diffuse, blanching, figured urticarial rash all over her body (Fig. 1). Non-pitting edema was also present on hands and feet, bilaterally. The joints were not painfull and no movement impairment was reported. Cardiovascular examination was normal, with normal sounds heard and no added sounds or murmurs. Chest examination was clear with bilateral air entry and no added sounds. Abdominal examination was also unremarkable: the abdomen was soft, without tenderness or organomegaly. Neurological examination was in the normal range. We performed a urine dipstick, that resulted normal. A dermatological evaluation described the clinical picture as a ‘non-specific urticarial rash’ and recommended antihistaminic drugs.

The following day, the abdominal pain and the urticarial rash worsened and she was conducted to the emergency department, where the previous dermatological diagnosis was confirmed, and she was discharged with antihistamines and corticosteroids.

Upon administration of the prescribed therapy, the rash disappeared and no additional abdominal pain occurred.

Three days after the disappearance of the rash, the patient, totally recovered, underwent the second administration of the Pfizer-BioNTech BNT16B2b2 mRNA vaccine.

**Fig. 2:**
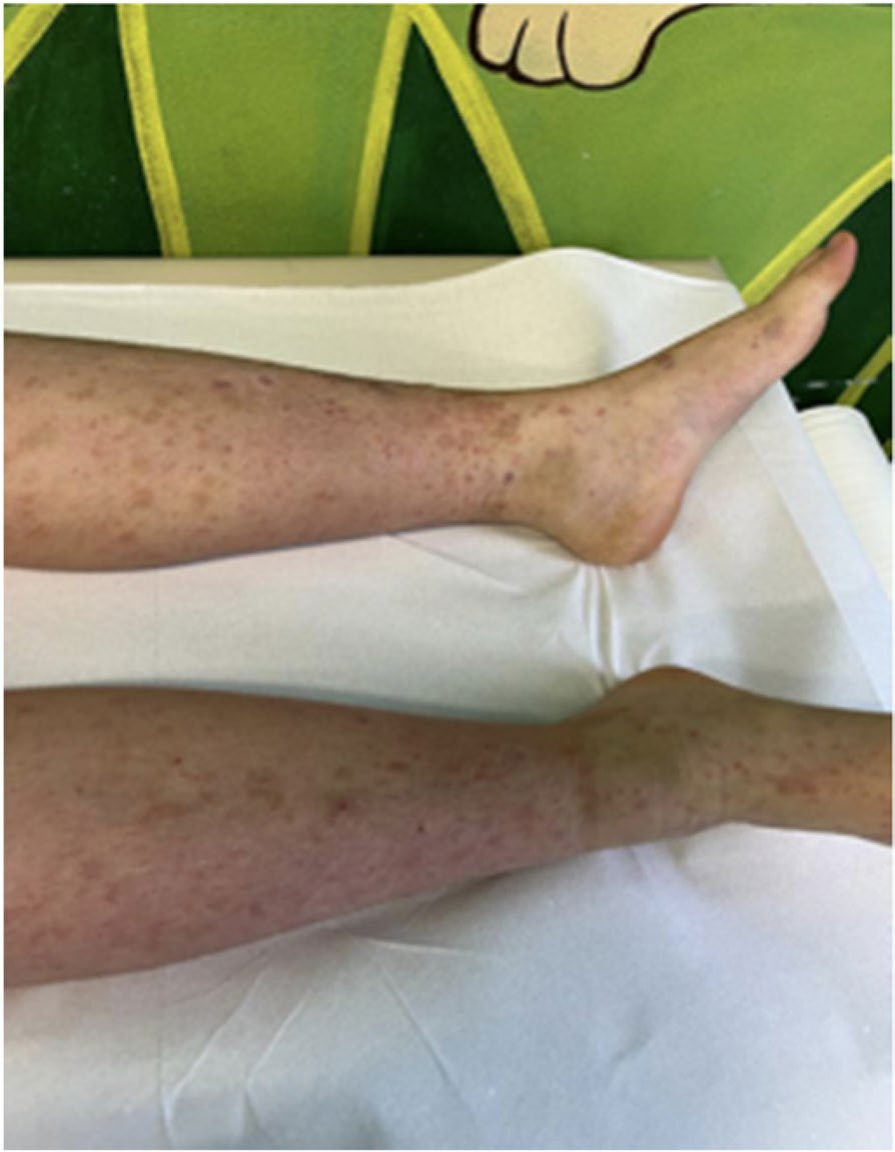
The rash became purpuric on her limbs

Five days later, the girl presented again to our evaluation with abdominal pain, one episode of vomiting and a new type of rash, mainly in her lower limbs and in her buttocks. The rash was slightly pruritic and purpuric. Physical examination revealed palpable, non-blanching petechiae and purpuric papules on lower limbs and at the bottom level (Fig. 2). Again, the objective examination and the urine dipstick were normal.

Indeed, during the following days, the rash got worse and the girl had severe attacks of abdominal pain, without diarrhea or vomiting, thus she was accompanied to the nearest hospital and admitted for further evaluations. Nasopharyngeal swab for Sars-Cov-2 and pharyngeal swab for Streptococcus group A were negative. Laboratory investigations revealed normal full blood count, normal electrolytes, normal liver and renal function tests, erythrocyte sedimentation rate and C reactive protein values as well as normal coagulation profile. An anti-streptolysin-O titre was also normal, as were the anti-nuclear antibody, anti-DNA antibody, anti-smooth muscle antibody and serum IgA, IgM. A slight increase in IgG (1516 mg/dL NV 690–1400 mg/dL), IgE (289 kU/L NV 0–120 kU/L) and anti-neutrophil cytoplasmic antibodies (atypical ANCA pattern) were reported (Table 2). Viral serology for Cytomegalovirus, Epstein Barr Virus and Mycoplasma Pneumoniae were negative for a current infection. Urine dipsticks, daily performed, were unremarkable. Moreover 2 out of 3 samples were positive for fecal occult blood testing.

**Table 2:** Patient’s laboratory bood examinations

	**Patient’s results**	**Normal Values**	**Units**
Leukocytes	6,5	4–10	10^3/μ L
Erythrocytes	4,68	3,83–5,08	10^6/μ L
Hemoglobin	12,9	12–16	g/dl
Platelets	263	140–400	10^3/μ L
C-reactive protein (CRP)	0,4	< 0,5	mg/dl
Erythrocyte Sedimentation Rate (ESR)	24	0–20	mm/h
IgG	**1516**	690–1400	mg/dl
IgA	171	70–400	mg/dl
IgM	115	40–230	mg/dl
IgE	**289**	0–120	kU/ml
C3	143	90–180	mg/dl
C4	28	10–40	mg/dl
Antistreptolysin O titer	167	< 150	UI/ml
Antinuclear antibodies (ANA)	Negative	-	-
Anti–smooth muscle antibodies (ASMA)	Negative	-	-
Anti–double-stranded DNA (dsDNA) antibodies	1,1	< 10	UI/ml
Anti-neutrophil cytoplasmic antibodies (ANCA)	**Positive**	-	-

During the hospitalization the rash improved, and she was diagnosed with vasculitis subsequent to Pfizer-BioNTech BNT16B2b2 mRNA vaccination. She was discharged with corticosteroid therapy (Prednisone 25 mg/day for 5 days) and both activity restriction and rest were recommended. Moreover, the adverse reaction was reported to the AIFA's National Pharmacovigilance Network (RNF).

**Fig. 3:**
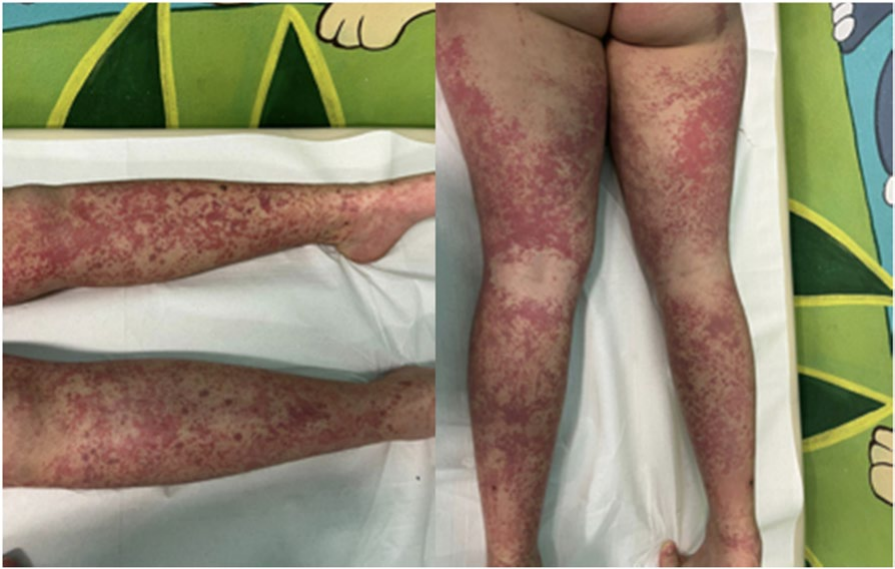
The purpuric rash spreading along the lower limbs after the second dose of Pfizer-BioNTech BNT16B2b2 mRNA vaccine

Two days after the rash worsened again and the purpuric eruption spread at the lower limbs and at the bottom (Fig. 3).

Again, the objective examination and the urine dipstick were normal. Thus, we urgently addressed her to a rheumathologist, who diagnosed a Henoch-Schönlein purpura associated with the Pfizer-BioNTech BNT16B2b2 mRNA vaccine and confirmed the ongoing corticosteroid therapy for 1 month.

Moreover, a new dermatological assessment, which led to cutaneous biopsy, was recommended. Histological examination and direct immunofluorescence study of the skin biopsy confirmed the clinical diagnosis of Henoch-Schönlein purpura showing vasculitis and IgA deposition along cutaneous vessels.

**Fig. 4:**
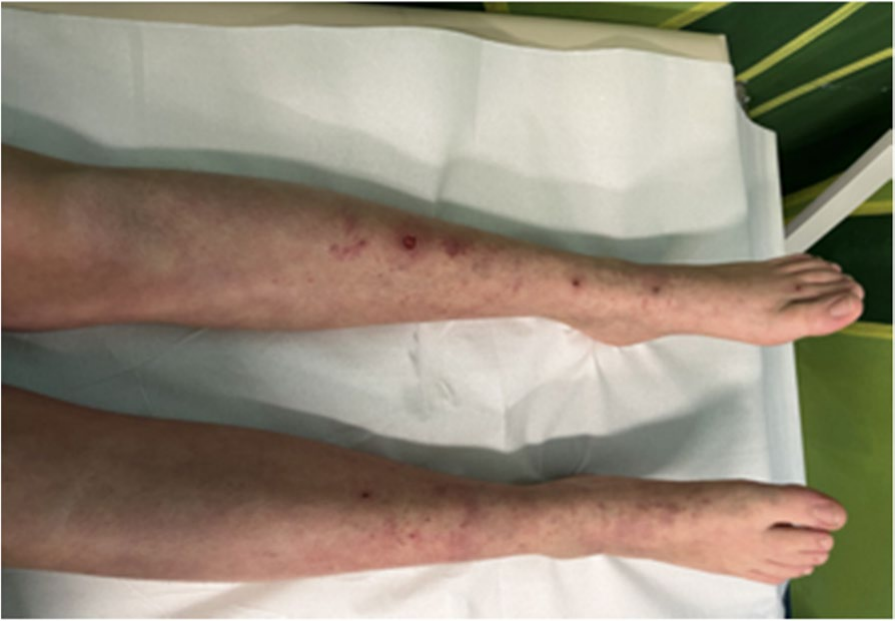
The rash progressively diminished after corticosteroid administration

In the following 15 days, upon corticosteroid administration, the rash progressively diminished (Fig. 4). Moreover, the objective evaluation and urine dipstick, performed weekly, were normal.

At the end of the 1-month corticosteroid therapy, the rash completely resolved.

At the last rheumatological visit, one month after the discontinuation of the steroid therapy, the patient appeared in good general condition. The purpuric lesions had regressed except for the persistence of an ulcerative lesion on the anterior surface of the left leg. There were no signs of arthritis on joint physical examination. The electrocardiogram and echocardiography performed at the cardiological visit were normal. Follow-up laboratory tests including complete blood count, c-reactive protein, erythrocyte sedimentation rate, antistreptolysin titre, electrolytes, liver and kidney function, and urinalysis were normal. The autoantibody panel that cytoplasmic antibodies (p-ANCA and c-ANCA), antinuclear antibodies, anti-DNA antibodies, anti-ENA antibodies, and anti-smooth muscle antibodies were also negative. Occult blood performed on a stool sample was negative.

This work was conducted after receiving the patient’s informed consent to publish this report and images.

## Discussion and conclusion

Our patient had the clinical signs and symptoms of HSP, fulfilling the EULAR/PRINTO/PRES criteria [2]. Indeed, she had both the mandatory criterion (palpable purpura in the absence of thrombocytopenia) and two of the supporting criteria: abdominal pain and typical histopathologic findings.

The most common triggering factor for HSP is a preceding upper respiratory tract infection, but the same condition was reported following vaccines [3–5]. The vaccine associated to HSP that is mostly studied in children is the measles-mumps-rubella (MMR) vaccine, with a three-fold increased risk of developing HSP associated with it [3]. Actually, different studies [2, 6, 7] reported HSP upon COVID-19 infection in children. Moreover, recently Hines et al. [8], Sirufo et al. [9] and Naithlo et al. [10] reported new onset HSP in adults following COVID-19 vaccine. Sirufo et al. describe a case of a 76 year-old female with coxalgia and episodes of microhematuria seven days after the first dose of the Vaxzevria (ChAdOx1 nCoV-19 AZD1222) vaccine [9]. Instead, Hines et al. presented a case of a 40-year-old female with a Henoch-Schönlein purpura twenty days after the second dose Pfizer-BioNTech BNT16B2b2 mRNA vaccine, characterized by a purpuric rash on gluteal region and macrohematuria, which was the first case of HSP after a mRNA vaccine [8]. Furthermore, Naithlo et al. reported a case of a 62-year old male with HSP 8 days after first dose of Oxford-AstraZeneca COVID19 = ChaAdOx1 nCoV-19 vaccine (AZD1222), with a renal and articular involvement [10]. HSP reactivation upon COVID-19 vaccination in a 45-year-old woman has been described [11], suggesting that autoimmune memory of vasculitis may persist and can be reactivated by COVID-19 vaccine. Interestingly, Cavalli et al. report three italian cases of cutaneous vasculitis and purpuric rash developing in previously healthy individuals shortly after vaccination with ChAdOx1 nCoV-19. Specifically, in the first patient, a 57-year-old man with a history of hypertension but no previous personal or family history of autoimmunity, a purpuric rash, initially affecting the lower limbs then rapidly spreading all over the body, developed around 14 days following the first vaccine dose; in the second patient, a 58-year-old man, with a silent medical history, purpura developed 7 days following the second dose of vaccine, spreading from the lower limbs to the abdomen and trunk; finally, in the third patient, a 53-year-old woman with no history of autoimmunity, purpura (mainly on the lower and upper limbs) developed 6 days following the first dose [12].

Other studies regarding the development of HSP after COVID-19 infection and COVID-19 vaccination are reported in the (Table 3).

**Table 3:** Studies regarding development of HSP following COVID-19 infection versus HSP following COVID-19 vaccination in adult and pediatric population

**HSP after COVID-19 infection**	**HSP after COVID-19 vaccination**
**Adult population:**	**Adult population:**
- Messova et al.: Covid-19 and new onset of IgA vasculitis: a systematic review of case reports - Jedlowski et al.: Coranavirus disease 2019-associated immunoglobulin A vasculitis/Henoch-Schonlein purpura: a case report and a review - Onate et al.: IgA vasculitis with nephritis(Henoch-Schonlein purpura) after COVID-19: a case series and review of the literature - Hashizume et al.: Immunoglobulin A vasculitis post-severe acute respiratory syndrome coronavirus 2 vaccination and review of reported cases	- Hines et al.: Henoch-Schönlein Purpura Presenting Post COVID-19 Vaccination - Sirufo et al.: Henoch-Schönlein Purpura Following the First Dose of COVID-19 Viral Vector Vaccine: A Case Report - Naitlho et al.: A Rare Case of Henoch-Schönlein Purpura Following a COVID-19 Vaccine—Case Report - Nishimura et al.: Vasculitis following COVID-19 vaccination - Nakatani et al.: New-Onset kidney biopsy-proven IgA vasculitis after receiving mRNA -1273 COVID-19 vaccine: case report - Muner et al.: De novo immunoglobulin A vasculitis following exposure to Sars-Cov-2 immunization - Maye et al.: Reactivation of IgA vasculitis following COVID-19 vaccination - Obeid et al.: Reactivation of IgA vasculitis after COVID-19 vaccination - Choi et al.: Sudden onset of IgA vasculitis affecting vital organs in adult patients following Sars-Cov-2 Vaccines - Sugita et al.: Development of Iga vasculitis with severe glomerulonephritis after Covid-19 vaccination: a case report and literature review - Valero et al.: Vasculitis flare after COVID-19: reports of two cases in patients with preexistent controlled Iga vasculitis and review of the literature - Ball-Burack et al.: A case of leukocytoclastic vasculitis following Sars-Cov-2 vaccination - Kondo et al.: Possible HSP reactivation post-COVID-19 vaccination and booster - Badier et al.: IgA vasculitis in adult patient following vaccination by ChadOx1 nCov-1 - Grossman et al.: Post-COVID-19 vaccination IgA vasculitis in an adult - Iwata et al.: Case of immunoglobulin A vasculitis following coronavirus disease 2019 vaccination - Abdelmaksoud et al. Sars-Cov-2 vaccination-induced cutaneous vasculitis: report of two new cases and literature review
**Pediatric Population:** - Asiri et al.: New-onset Henoch-Schonlein Purpura After COVID-19 infection: A case report and a review of the literature - Messova et al.: Covid-19 and new onset of IgA vasculitis: a systematic review of case reports - El Hasbani et al.: Henoch-Schonlein purpura: another COVID-19 complication - Borocco et al.: Sars-Cov-2-associated Henoch-Schonlein purpura in a 13-year-old girl - Serafinelli et al.: Kidney involvement and histological findings in two pediatric COVID-19 patients - Hashizume et al.: Immunoglobulin A vasculitis post-severe acute respiratory syndrome coronavirus 2 vaccination and review of reported cases	**Pediatric Population:** - Casini et al.: Henoch-Schonlein purpura following COVID-19 vaccine in a child: a case report

This case of classic HSP as an adverse reaction Pfizer-BioNTech BNT16B2b2 mRNA vaccine is the first such report in pediatric patients, to the best of our knowledge. The exact pathophysiological mechanisms behind the vasculitis appearance upon COVID-19 vaccine administration is still not fully understood. The presence of HSP both in SARS-CoV-2 positive patients [2, 6, 7] and following vaccination [8–10] suggests a similar underlying etiology of immune activation. The main hypothesis considers the reaction an immunological process, triggered by vaccine antigen (in this case the spike protein) forming immune complexes which, depositing on vessel walls, induce inflammation and immune system hyperactivation [13].

Similarly, Chen et al. reported that also multisystemic inflammatory syndrome in children (MIS-C) associated to COVID-infection could be a result of an antigenic exposure that triggers a production of inflammation-inducing substances in genetically susceptible patients, particularly in association with HLA B*46:01 and HLA B*15:03. However, based on the genetic predisposition, the clinical manifestations and the severity may be various and heterogeneous. [14].

Indeed, an immune-mediated etiology is plausible in our patient, as the skin rash was recurrent and was notably more severe following the second injected dose, possibly for further enhancement of the immune response. Importantly, in the reported patient no further “booster dose” was required but, if the girl had been older, this issue should have been addressed. Subsequent administration of the vaccine could potentially trigger a furthermore severe immunologic reaction, as shown by the increasing severity of the rash occurring after the second administration. There are currently no clear guidelines for management of revaccination in patients with a history of vaccine-induced vasculitis [15], thus, concerning this topic, further evaluations are needed and a deep risk–benefit evaluation should be done.

Our case highlights the probable association between HSP and Pfizer-BioNTech BNT16B2b2 mRNA vaccination. Based on Adverse Event Following Immunization (AEFI) WHO causality assessment tool, the observed event has a consistent causal association to immunization (absence of strong evidence for another cause, evidence for the association in the literature, temporal association with the vaccine administration), and can be considered a vaccine product-related reaction [16].

Nonetheless, the benefits of vaccination greatly outweigh the risks. When millions of individuals are vaccinated during a world-wide immunization campaign, even rare immune-mediated adverse events, as the inflammatory response in predisposed individuals, may be observed. The reports of vasculitis, especially in the pediatric age, are rare if compared to the millions of doses of vaccine administered; in Italy, 5 millions children and adolescents have been vaccinated so far [12, 17].

However, clinicians should be aware of this possible complication if a patient presents symptoms correlated with HSP after COVID-19 vaccination, to correctly address the patient and perform all the investigations required. For this reason, we also reported the suspected adverse reaction to the AIFA's National Pharmacovigilance Network (RNF) and further studies are needed to confirm this relationship and elucidate the mechanisms behind this phenomenon, especially in the pediatric population.

## Data Availability

All data generated or analyzed during this study are included in this published article.

## Abbreviation

HSP: Henoch-Schönlein purpura
